# How I Can Suspect of Mycobacteria Infection in Breast Implant Surgery?

**Published:** 2016-09

**Authors:** Guillermo Ramos-Gallardo

**Affiliations:** Vallarta Medical Center, Jalisco, México

**Keywords:** Breast implant, Mycobacteria, Infection

**DEAR EDITOR**

Infection is a possible complication in breast implant surgery especially during the first six months after surgery.^1^ In 2014, Committee of Security of the Mexican Society of Plastic, Aesthetic and Reconstructive Surgery published the results of a survey about Infections in Breast Implants in the society members. The most common organism involved were *Staphylococcus*, especially species *S. aureus* and *S. epidermidis*. Other type of bacteria were reported as *Pseudomonas*, E*. coli*, *Streptococcus*, *Enterobacter*, *Klebsiella* and *Mycobacteria*.^1^ The symptoms, diagnoses and treatment of non-tuberculous or atypical *Mycobacteria* in breast implants were previously reported.^2^ In one survey, 6.5% of the surgeons reported at least one case of *Mycobacteria*.^3^ *M. xenopi* usually causes lung disease. There are some reports about wrist tenosivitis or soft tissue infection.^3^ The following case represents the first know report of *M.* *xenopi* as cause of breast implant infection.

A 53 years old female consult for breast asymmetry after breast augmentation eight years ago. At the time of presentation, she denied any pain, swelling, erythema or fever. Preoperative ultrasound and mammogram were reviewed before surgery. No malignancy was reported. In right side (the bigger side), fluid around the implant was identified. Surgery was scheduled as revision surgery to correct breast asymmetry and ptosis. In the preoperative consult, we discussed surgery, risk and complications. Patient denied the possibility to remove breast implants. 

During the surgery white and odorless fluid was aspirated from the right side. Fluid samples were sent to pathology and microbiology. Figure 1 shows view of capsule at time of first surgery. Results were negative to identify any possible cause, including gram stain, cultures, Sabouraud and Lowestein. Cultures for *Mycobacteria* were found negative. Pathology reported chronic inflammation and fibrosis. The postoperative period evolved without any anomaly during the first week. After 3 months, the patient reported leakage of white fluid from the lower border of the inframammary fold of the affected breast. At this time PCR was requested to identify the organism. The result was positive for *M. xenopi*. Bilateral breast implant removal and capsulectomy were accepted to be undertaken for the patient. Treatment with etambutol, rifampicin and pyrazinamide was initiated for six months. 

**Fig. 1:**
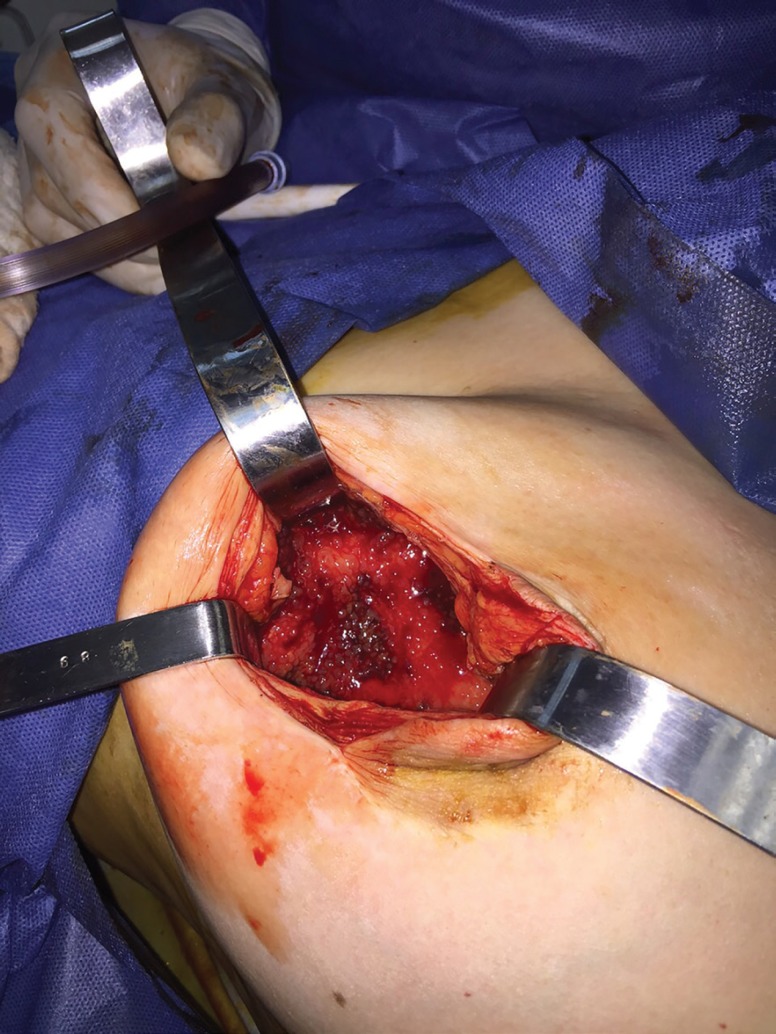
The presence of a capsule in breast tissue during surgery

In the literature, most of the cases that involve *Mycobacteria* infection in breast implants, reported the presence of the subtype *M. fortituim*. This is the first case know of the subtype *M. xenopi*. Report cases of breast implants and infection caused by *Mycobacteria *have been from different countries, such as Israel, Canada, United States, India and Brazil.^4^^-^^8^ In one of the reports, they identified in the beard of the surgeon, the presence of *Mycobacteria *that was isolated from the whirlpool bath. One of the possible causes of the outbreaks could be water supply of the hospitals.^4^^-^^8^ Other important factor is biofilm presence because bacteria can live inactive and later in appropriate environment cause diseases. This is the possible explanation of a case that involved a tattoo and *Mycobacteria* infection. Ink was suspected the cause of *Mycobacteria *infection in breast implants in a patient that had a tattoo in the back and planned to do breast reconstruction.^9^

In this case, especial attention can be given on two points. First, denial of the patient to remove breast implants. Probably it is motivated by the relation of breast and beauty and the connection to self-esteem. Second, most of the time the *Mycobacteria* infection is evident during the first 30 days following surgery.^10^ The patient came to clinic requesting correction of breast asymmetry. Symptoms as fever, pain, swelling or erythema were not evident. Plans were done to correct breast asymmetry and ptosis. The finding of white, odorless fluid was not expected at that moment. 

First report of a *Mycobacteria* outbreak was done in 1983 who identified 17 patients with infection of breast implants as *M. fortituim*. All implants were removed and patients started during the early postoperative period with symptoms.^4^ In Israel, after ruling out any source inside the hospital (operating rooms, air conditioner or water supply), cultures from medical team were checked. It is interesting to find out that bacteria was in the beard and moustache of the surgeon. Bacteria especially *Mycobacteria *can live in inanimate surface as whirlpool bath.^5^ Hand washing is imperative to prevent any transmission from doctors or nurses to patients but as in this case was not enough to prevent infection.^5^

Regarding 80 cases in the literature, the median age was 34.53 years (15–70 years). Two patients reported comorbidities of lupus and diabetes mellitus. In 31 patients, the side affected, 14 bilateral, 10 left and 7 right areas. In 32 patients, symptoms started during the first month after surgery. Most common symptoms were erythema and swelling in 43 cases plus fluid from the surgical incision in 35 and fever in 2 more patients. The reason of breast implant was mentioned in 68 patients, where 55 were aesthetic and 13 were reconstructive.^5^^-^^8^^,^^10^^-^^14^

Diagnoses were done with cultures in 49 patients and it was possible to identify *M. genoma* in 32. Most common was *M. fortuitum* in 39, *M. jacuzzi* in 14, non-specified in 14, *M. abscessus* in 4, *M. avium* in 3 and with only one case report of *chelonae*, *conceptionense*, *goodi*, *parafortuitum*, tuberculosis and *porcinum*. In 22 patients, empiric antibiotics were started without knowing results of the microorganism involve. Most common treatment included two or more antibiotics such as combination of ciprofloxacin, amikacin and clarytromicin in 14 cases, ciprofloxacin and gatifloxacyn in 12 cases and ciprofloxacin and doxiciclyn in 7 cases. Treatment lasted for 13 weeks (3 to 30 weeks). Three cases did not receive antibiotics.^5^^-^^8^^,^^10^^-^^14^

Most of the cases were outbreaks and the possible source was water supplied from cities or hospitals. This is the first case report with *M. xenopi*. *Mycobacteria* can be divided in tuberculous and non-tuberculous. Non tuberculous are considered atypical *Mycobacteria*. *M. xenopi* is atypical *Mycobacteria* that was presented mainly in lung infections. Some reports mentioned cases of tenosivitis or soft tissue infection in immunosuppressed patients.^3^ As we know, this is the first case of *M. xenopi* in breast implant infection. 

Most of the reports in literature showed symptoms during the first weeks after surgery. For this case, when the infection happened is not clear, probably during the first weeks after surgery and bacteria coexisted with patient (biofilm) or they were from lung infection and later was attracted by the breast implant. The patient did not show any lung disease and symptoms. PPD was not done because it could be positive through breast infection. Her family members were PPD negative. This is a case of *Mycobacterica* infection in breast implant, first case report with *M. xenopi*. 

This case scenario started with breast asymmetry where no fever, swelling, erythema or fluid was present at the beginning. Patient denied initially breast implant removal and no source of infection was found in this case. Therefore, as *Mycobacteria* can appear as outbreak in population probably hospital community where source of infection can be in unimaginable places as surgeon beard and sometimes in immunocompromised patients and mostly during the first days after surgery and there is no conventional treatment, especial attention should be paid for these cases.

## CONFLICT OF INTEREST

The authors declare no conflict of interest.
